# Silver Nanoparticles Enhance the Antibacterial Effect of Antibiotic-Loaded Bone Cement

**DOI:** 10.7759/cureus.34992

**Published:** 2023-02-14

**Authors:** Lokman Kehribar, Mahmud Aydın, Hüseyin Sina Coşkun, Serkan Surucu

**Affiliations:** 1 Orthopedics and Traumatology, Samsun University, Samsun, TUR; 2 Orthopedics and Traumatology, Haseki Education and Research Hospital, Istanbul, TUR; 3 Orthopedics and Traumatology, Samsun Ondokuz Mayis University Faculty of Medicine, Samsun, TUR; 4 Orthopedics and Rehabilitation, Yale University, New Haven, USA

**Keywords:** nanoparticles, polymethyl methacrylate (pmma), silver nanoparticles, bone cement, antibacterial activity

## Abstract

Purpose

The goal of this study was to determine the antibacterial activity of bone cement in polymethyl methacrylate (PMMA) structures with varying amounts of silver nanoparticles (AgNPs) included. Additionally, we aimed to evaluate whether AgNPs affect the biomechanical properties of PMMA cement in our study.

Materials and methods

Between April 2020 and June 2020, we conducted a series of experiments to demonstrate the antibacterial characteristics by adding silver nanoparticles to PMMA bone cement. PMMA bone cement (Cemex, Tecres Company, Verona, Italy) was used as the base material. Seven different samples were prepared in order to evaluate the amount and presence of AgNPs. Cement samples containing AgNPs and teicoplanin at different concentrations and empty cement (control, without teicoplanin and AgNPs) were placed on Petri plates. The agar diffusion method was used to determine the antibacterial effect (Kirby-Bauer).

Results

Kirby-Bauer assays demonstrated that AgNPs added to bone cement increased the antimicrobial activity compared to antibiotic-free or only teicoplanin-loaded cement. It was observed that increasing the AgNPs ratio further increased the antimicrobial activity.

Conclusion

AgNPs in various combinations enhance antimicrobial activity synergistically while maintaining the mechanical strength of bone cement. Increasing the amount of AgNPs results in a significant increase in antimicrobial activity.

## Introduction

Bone cement (BC) is a biomaterial that is routinely used during orthopedic surgeries [1]. Today, BC is used to fix prostheses, fill bone defects and dead spaces, treat bone infections, and provide vertebral stability [2]. Increased life expectancy, rising rates of osteoporotic and pathological fractures, and an increase in the number of arthroplasty procedures have all contributed to an increase in the use of bone cement [3]. While there are numerous types of BC with varying structures and qualities, polymethyl methacrylate (PMMA) is often utilized in orthopedics due to its ease of production, adequate mechanical properties, and biostability in the human body [4].

With the growing usage of BC in clinical practice, numerous modifications have been produced. One of these modifications is the introduction of antibiotics into the cementum to avoid bacterial infection during PMMA implantation and to provide an antibacterial effect during infection treatment [5]. Antibiotics such as gentamicin and vancomycin have been added to BC to achieve an antibacterial effect in the treatment of specific infections [6]. However, since the use of antibiotics can lead to the development of resistance and the duration of action is limited, it has been discussed by some authors, and different studies have emerged. The use of silver, a broad-spectrum antimicrobial agent with low toxicity, is one of them [7]. While silver can be utilized in a variety of ways, its utilization in the form of nanoparticles provides several advantages [8]. The primary advantages of silver nanoparticles (AgNPs) are their low toxicity and prolonged effect. Simultaneously, the acidic environment created by adhering bacteria might accelerate the rate of release [9].

The purpose of this study was to determine the antibacterial activity of BC in PMMA structures with varying amounts of AgNPs included. We aimed to evaluate whether AgNPs affect the biomechanical properties of PMMA cement in this study.

## Materials and methods

Bone cement preparation

**Figure 1:**
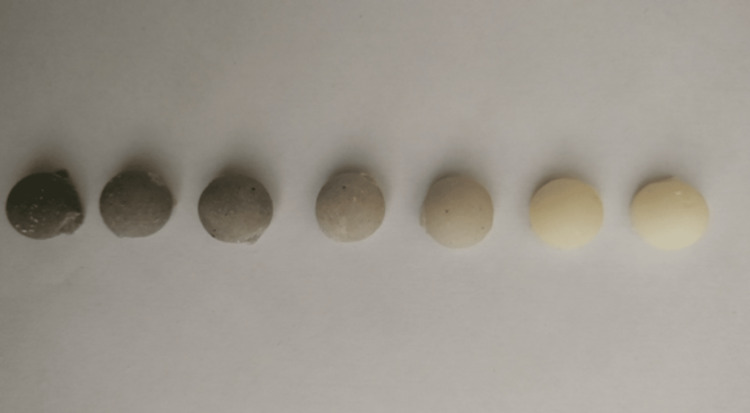
The preparation of teicoplanin and AgNPs in various combinations with PMMA AgNPs: Silver nanoparticles; PMMA: Polymethyl methacrylate.

This study was conducted to show that by adding silver nanoparticles to PMMA BC, it acquires antibacterial properties. This study was carried out between April 2020 and June 2020. PMMA BC (Cemex, Tecres Company, Verona, Italy) was used as the base material. Then, seven different samples were prepared in order to evaluate the amount and presence of AgNPs. A commercial monomer containing 15 g of cement and 5.5 mL of initiator was used for each group. The ratio of teicoplanin/cement as a model drug was adjusted to 40/400 mg according to the supplier's procedure. The effects of AgNPs were investigated by keeping the amount of cement and antibiotics constant in the groups. The silver nanoparticles (PMMA-AgNPs) were mixed with cement homogeneously with 10, 7.5, 5, 2.5, and 1.25 mg of silver nanoparticles (30 nm in diameter and purity 99.9%), then an initiator was added, and polymerization was achieved by filling into silicone molds immediately after obtaining a homogeneous paste (Figure 1).

BC samples were prepared following the manufacturer's instructions and according to the international standard ISO 5833:2002. Empty cement samples containing only teicoplanin were also prepared for control purposes. Experimental groups were organized for bacterial activity. The applied contents of modifiers and BC components are presented in Table 1.

**Table 1:** Bone cement composition based on the concentration of AgNPs BC: Bone cement; PMMA: Polymethyl methacrylate; AgNPs: Silver nanoparticles. *For statistical purposes, the experiment was repeated three times for each group.

BC with nanoparticle	Experimental group*
BC-10 mg AgNPs	PMMA, 10 mg AgNPs and 50 mg teicoplanin
BC-7.5 mg AgNPs	PMMA, 7.5 mg AgNPs and 50 mg teicoplanin
BC-5 mg AgNPs	PMMA, 5 mg AgNPs and 50 mg teicoplanin
BC-2.5 mg AgNPs	PMMA, 2.5 mg AgNPs and 50 mg teicoplanin
BC-1.25 mg AgNPs	PMMA, 1.25 mg AgNPs and 50 mg teicoplanin
BC only teicoplanin	PMMA and 50 mg teicoplanin

Antimicrobial activity

**Figure 2:**
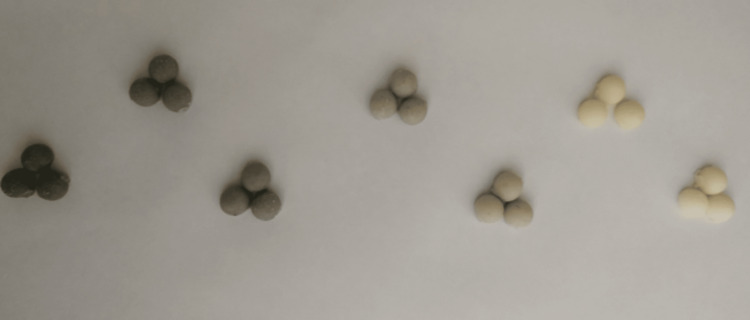
Preparing three different samples from each experimental group. The concentration of AgNPs is higher in the dark black samples. AgNPs: Silver nanoparticles.

The agar diffusion method was used to determine the antibacterial effect (Kirby-Bauer). This method is based on the detection of bacterial growth inhibition. Evaluation is made by measuring the inhibition zone diameter formed. At this stage of the study, the aim was to investigate the antibacterial effects of BC samples containing silver nanoparticles and teicoplanin at different concentrations on *Staphylococcus aureus* (*S. aureus*) and *Enterococcus faecalis* (*E. faecalis*) microorganisms. For this purpose, these microorganisms were inoculated into sterile Luria Bertani (LB; tryptone [1.0%], yeast extract [0.5%], and NaCl [1.0%]) broth. Then, overnight cultures were prepared by incubating at 37°C at 150 rpm for 18 hours in a shaking oven. After the culture suspensions of *S. aureus* and *E. faecalis* strains in the logarithmic growth phase were adjusted according to the 0.5 Mc Farland standard (1.5 x 10^8^ CFU/mL), 100 µl of bacterial suspensions were sampled and inoculated separately on Mueller-Hinton agar medium with a sterile swab. Then, cement samples containing AgNPs and teicoplanin at different concentrations and empty cement (negative control) were placed on Petri plates. After the Petri dishes were incubated at 37°C for 24 hours in a static oven, the diameters of the inhibition zones were measured to determine antimicrobial susceptibility. These experiments were repeated three times (Figure 2).

Biomechanical testing

The mechanical properties were evaluated via a compression test with a Zwick Z010 universal testing machine (Kennesaw, GA, USA). The spherical samples were placed in the sample holder of the device. Time to fracture according to the forces applied to fracture was recorded under strain parameters as preloading force, force rate, and maximum threshold force to finish experiments were 0.1 N, 5 mm/min, and 10%, respectively. The measurements were repeated separately for three samples.

## Results

Antimicrobial activity

Results obtained from the Kirby-Bauer assays demonstrated that AgNPs added to BC increased the antimicrobial activity compared to antibiotic-free or only teicoplanin-loaded cement. Furthermore, it was observed that increasing the AgNPs ratio further increased the antimicrobial activity. BC with 10 mg AgNPs exhibited large zones of inhibition (32.6 and 30.3 mm, respectively) for an isolate of *S. aureus *and *E. faecalis*. Detailed zone diameter measurements for seven different experimental groups are summarized in Table 2.

**Table 2:** Resulting zone of inhibition from the Kirby-Bauer assays at different bone cement ingredients against isolate strains of S. aureus and E. faecalis BC: Bone cement; AgNPs: Silver nanoparticles.

	S. aureus			E. faecalis		
BC with nanoparticle	Mean (mm)	Median	Standard deviation	Mean (mm)	Median	Standard deviation
BC-10 mg AgNPs	32.6	32	4	30.3	29	5.3
BC-7.5 mg AgNPs	21.3	21	0.6	30.2	28.5	5
BC-5 mg AgNPs	20.3	20	1.5	28	28.5	1.5
BC-2.5 mg AgNPs	19,7	20	1.5	27	26	3.6
BC-1.25 mg AgNPs	17.3	17	1.5	25.3	26	3.1
BC only teicoplanin	16.3	16	0.6	25.7	24	3.8
Only BC	14	14	1	19	20	2.6

Biomechanical testing

**Figure 3:**
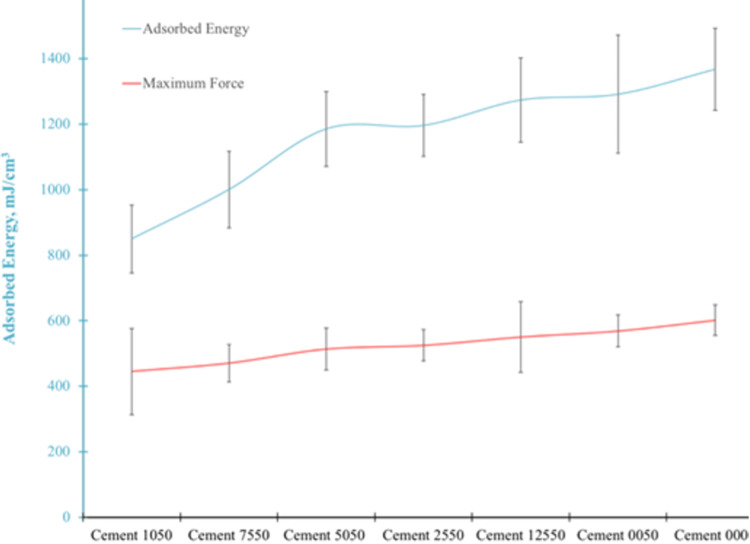
Compression tests on AgNP-loaded bone cements in various concentrations Code 1050 of the cement series contained 10 mg AgNPs and 50 mg Targocid; code 7550 contained 7.5 mg AgNPs and 50 mg Targocid; code 5050 contained 5 mg AgNPs and 50 mg Targocid; code 2550 contained 2.5 mg AgNPs and 50 mg Targocid; and code 0050 contained 0 mg AgNP and 50 mg Targocid. Code 0000 for the cement series did not contain AgNP and Targocid. AgNPs: Silver nanoparticles.

The higher AgNP loading into PMMA resulted in a shift to lower absorbed energy that revealed the increase in the structural fragility of the PMMA. On the other hand, the AgNP-loaded BC in various concentrations showed a similar maximum force durability. The effect of the AgNPs concentration on the compressibility of the PMMA is shown in Figure 3.

## Discussion

The study's most significant finding is that when teicoplanin and AgNPs are combined in various combinations, they synergistically enhance antimicrobial activity while preserving the mechanical strength of BC. Additionally, the study demonstrated that increasing the number of AgNPs increases antibacterial activity significantly.

AgNPs are used in a wide range of applications, including medical imaging, drug administration, dental materials, nanocomposites, antibacterial agents, and bone cement [10]. AgNPs have recently generated considerable interest in biomedical applications due to their antibacterial efficacy and non-toxic nature. Although numerous investigations have demonstrated their antibacterial activity, the exact mechanism remains unknown. Various theories have been suggested on the effect of AgNPs. This effect has been attributed to either the direct release of silver ions or the indirect production of free radicals by AgNPs [7]. Silver ions or free radicals can interact with bacterial cells, causing damage to the cell membrane or inhibiting critical enzyme activities [11,12]. Additionally, AgNPs can disrupt signal transduction, thus preventing bacterial growth [12].

Antibiotic-loaded BCs are also frequently used in orthopedic procedures, especially in revision surgeries. BC is biocompatible as polymerization is very simple and adaptable for quick application in operating room conditions. On the other hand, the low porosity of the BC may limit antibiotic release, and increasing the temperature during polymerization causes denaturation of the antibiotic structure. Furthermore, increasing the number of antibiotics leads to a reduction in the mechanical strength of BC. Finally, in antibiotic-resistant infections, antibiotics alone may not be sufficient. To eliminate these problems, AgNPs appear to be an attractive alternative to conventional antibiotics.

The evaluation of the antibacterial effect of AgNPs-loaded BC was conducted in 2004 [13]. Alt et al. [13] studied the in vitro antibacterial activity against *Staphylococcus epidermidis*, methicillin-resistant *S. epidermidis *(MRSE), and methicillin-resistant *S. aureus* (MRSA). While plain cement and gentamicin-loaded cement were ineffective against MRSA and MRSE, AgNP-loaded cement demonstrated strong antibacterial activity. Similarly, Wekwejt et al. [14] reported that BC modified with AgNPs revealed greater bactericidal effects and prevented biofilm formation better compared to antibiotic-loaded BC. Likewise, Slane et al. [15] showed that AgNP-loaded BC reduced the biofilm formation on the surface of the cement. The authors also suggested that AgNP-loaded cement could be useful in primary arthroplasty.

The synergistic effect of AgNPs with antibiotics has been studied in several studies [16,17]. The possible mechanism may be related to the increase in cell wall penetration by antibiotics with AgNPs. Singh et al. [18] investigated the effect of the combination of silver nanoparticles with different commercial antibiotics to determine its activity against six pathogenic bacteria such as *Vibrio parahaemolyticus*, *Salmonella enterica*, *S. aureus*, *Bacillus anthracis*, *Bacillus cereus*, *Escherichia coli*, and *Candida albicans*. They discovered that anisotropic AgNPs improve antibiotic antimicrobial activity. Similarly, Naqvi et al. [19] reported that the synergistic effect of antibiotics and nanoparticles resulted in a mean 2.8-fold area increase in antibacterial activity. Our study also showed that antibiotic-loaded PMMA with 10 mg AgNPs exhibited large zones of inhibition, which clearly reveals that AgNPs can be effectively used in combination with antibiotics in order to improve their efficacy against an isolate of *S. aureus* and *E. faecalis*. Based on these results, we suggest that the combination of AgNPs with commercial antibiotic-loaded PMMA may be performed in revision surgeries against various pathogenic-resistant microbes.

The mechanical strength of antibiotic-loaded BC is another concern as this can lead to a reduction in the compressive strength in vitro [5]. The addition of 2 g of antibiotic to a 40 g PMMA mixture has no effect on short-term mechanical properties [20]. Slane et al. [15] investigated the effect of the addition of AgNPs on the mechanical properties of BC. They reported that the material properties were not substantially different from those of standard cement. Our study also showed that the AgNPs loaded into bone cement in various concentrations showed a similar maximum force durability.

The present study has several limitations. First, a single antibiotic (teicoplanin)-loaded PMMA was used, and the effect of the AgNPs as an additive in various concentrations was evaluated. It is possible that the other types of antibiotics could reveal different results. Second, only two bacterial strains (*S. aureus* and *E. faecalis*) were used for antibacterial testing, but others could respond differently to the AgNPs loaded in PMMA. Third, the potential toxic effect of the AgNPs was neglected, which could be in over-concentration. The threshold value for the AgNP concentration leading to damage in human cells is still unknown [21]. Further in vivo and in vitro studies are necessary to support the findings as definitive conclusions.

## Conclusions

AgNPs in various combinations enhance antimicrobial activity synergistically while maintaining the mechanical strength of bone cement. Increasing the amount of AgNPs results in a significant increase in antimicrobial activity.
